# Evidence for peripheral neuroinflammation after acute whiplash

**DOI:** 10.1097/j.pain.0000000000003560

**Published:** 2025-03-04

**Authors:** Colette Ridehalgh, Joel Fundaun, Stephen Bremner, Mara Cercignani, Soraya Koushesh, Rupert Young, Alex Novak, Jane Greening, Annina B. Schmid, Andrew Dilley

**Affiliations:** a Department of Clinical Neuroscience, Brighton and Sussex Medical School, Trafford Centre, University of Sussex, Falmer, Brighton, United Kingdom; b School of Life Course and Population Sciences, Faculty of Life Sciences & Medicine, King's College London, London, United Kingdom; c Nuffield Department of Clinical Neurosciences, John Radcliffe Hospital, University of Oxford, Oxford, United Kingdom; d Department of Anesthesiology, Perioperative and Pain Medicine, Stanford University School of Medicine, Palo Alto, CA, United States; e Department of Primary Care and Public Health, Brighton and Sussex Medical School, Brighton, United Kingdom; f Cardiff University Brain Research Imaging Centre, Cardiff University, Cardiff, United Kingdom; g School of Engineering and Informatics, University of Sussex, Brighton, United Kingdom; h Emergency Medicine Research Oxford (EMROx), Oxford University Hospitals NHS Foundation Trust, Oxford, United Kingdom

**Keywords:** Whiplash, Whiplash associated disorder, Neuroinflammation, Neuritis, Magnetic resonance imaging, Quantitative sensory testing, Heightened nerve mechanosensitivity, Neuropathic pain

## Abstract

Whiplash injury is associated with high socioeconomic costs and poor prognosis. Most people are classified as having whiplash-associated disorder grade II (WADII), with neck complaints and musculoskeletal signs, in the absence of frank neurological signs. However, evidence suggests that there is a subgroup with underlying nerve involvement in WADII, such as peripheral neuroinflammation. This study aimed to investigate the presence of neuroinflammation in acute WADII using T2-weighted magnetic resonance imaging of the brachial plexus, dorsal root ganglia and median nerve, and clinical surrogates of neuroinflammation: heightened nerve mechanosensitivity (HNM), raised serum inflammatory mediators, and somatosensory hyperalgesia. One hundred twenty-two WADII participants within 4 weeks of whiplash and 43 healthy controls (HCs) were recruited. Magnetic resonance imaging T2 signal ratio was increased in the C5 root of the brachial plexus and the C5-C8 dorsal root ganglia in WADII participants compared with HCs but not in the distal median nerve trunk. Fifty-five percent of WADII participants had signs of HNM. Inflammatory mediators were also raised compared with HCs, and 47% of WADII participants had somatosensory changes on quantitative sensory testing. In those WADII individuals with HNM, there was hyperalgesia to cold and pressure and an increased proportion of neuropathic pain. Many people with WADII had multiple indicators of neuroinflammation. Overall, our results present a complex phenotypic profile for acute WADII and provide evidence suggestive of peripheral neuroinflammation in a subgroup of individuals. The results suggest that there is a need to reconsider the management of people with WADII.

## Abstract 2

Supplemental Digital Content is Available in the Text.

This study demonstrated changes in magnetic resonance neurography and clinical measures indicative of peripheral neuroinflammation in people with whiplash-associated disorder grade II.

## 1. Introduction

Whiplash injury is associated with significant social and economic cost. In the United Kingdom alone, the cost of whiplash has been estimated to be £3 billion annually.^30^ Prognosis remains poor with approximately 50% of people living with pain and disability 5 years after injury.^59^ Following whiplash injury, most individuals are classified as having whiplash-associated disorder grade II (WADII) as defined by the Quebec Task Force.^53^ Whiplash-associated disorder grade II is defined as neck complaints accompanied by musculoskeletal signs, such as reduced range of movement and point tenderness^55^ in the absence of frank neurological signs (decreased/absent deep tendon reflexes, weakness, and sensory deficits). However, the broad definition of WADII has faced scrutiny because studies have identified sensory deficits in people classified under WADII, such as reduced vibration thresholds and thermal hypoesthesia.^20^ Moreover, following whiplash injury, some have signs of heightened nerve trunk mechanosensitivity, which include pain in response to both palpation over nerve trunks and tests, which apply tensile loads to upper limb nerves.^25^ Taken together, these findings suggest that some people with WADII have an underlying nerve pathology. Although the characteristics of such pathology are unclear, one likely contributing factor is peripheral neuroinflammation.

Much of the evidence for a role for peripheral neuroinflammation in musculoskeletal pain conditions comes from an animal model of neuritis. A significant feature of this model is the presence of a gain of cutaneous sensory nerve function, whereby animals exhibit both mechanical and thermal evoked cutaneous hypersensitivities, in the absence of axonal degeneration.^6,15,42,48^ Furthermore, apparent uninjured C-fibre nociceptors develop spontaneous activity and become mechanically sensitive to stretch and pressure at the inflammatory site.^6,12,13,22^ Such inflammation-induced axonal mechanical sensitivity is particularly interesting because it is consistent with the presence of heightened nerve mechanosensitivity (HNM) reported in those with WADII.^25^

Peripheral nerve pathology, such as neuroinflammation, can be visualised using magnetic resonance imaging (MRI).^2,18,52^ For example, animal studies have shown that an increased T2-weighted signal provides an indicator of intraneural oedema associated inflammatory changes.^5,54^ We have previously found preliminary evidence for increased T2-weighted signal in the roots of the brachial plexus in individuals with chronic whiplash, consistent with the presence of peripheral neuroinflammation.^23^ However, peripheral neuroinflammation has not been examined acutely following whiplash injuries.

The primary aim of this exploratory study is to identify evidence for the presence of peripheral neuroinflammation in acute WADII, which may be important for the future development of targeted therapies. We focused on increased T2-weighted MRI of the brachial plexus, dorsal root ganglia (DRG), and median nerve at the wrist, as a primary measure of neuroinflammation, as well as 3 clinical surrogates of neuroinflammation: presence of heightened nerve trunk mechanosensitivity of the upper limbs, raised levels of serum inflammatory mediators, and the presence of somatosensory hyperalgesia on both bedside neurological and quantitative sensory testing (QST). Overall, our results present a complex phenotypic profile for acute WADII and provide evidence suggestive of peripheral neuroinflammation in a subgroup of individuals.

## 2. Methods

### 2.1. Protocol registration

This article reports the baseline findings of a prognostic study prospectively registered at ClinicalTrials.gov (reference number NCT04940923; protocol version V2 25/9/20). The full study protocol was published in 2022.^44^

### 2.2. Ethical approval

Ethical approval was received from London-Brighton and Sussex Research Ethics Committee (20/PR/0625) and South Central—Oxford C Ethics Committee (18/SC/0263), and all participants provided informed written consent before participating.

### 2.3. Participants

Participants aged 18 to 60 years were recruited within 4 weeks following a whiplash injury from the Emergency Departments of 2 National Health Service (NHS) Trusts in the Southeast of England. Potential participants were contacted by a research nurse within the Trusts following weekly hospital database searches using defined search terms (eg, motor vehicle collision, muscle injury: neck, bruise/contusion/abrasion: neck, sprain/ligament injury: cervical spine). Potential participants were asked for permission for their details to be passed on to the study team and/or sent the study details. An investigator then screened any interested people for suitability via telephone. Individuals who met the Quebec Task Force classification of WADII (neck complaints and musculoskeletal signs, including decreased range of motion [ROM] and point tenderness in the neck) and who were eligible for the study were sent a participant information sheet and a link to complete questionnaires and were invited to attend an appointment. Written informed consent was obtained by a member of the study team at the appointment.

Exclusion criteria included pregnancy, recent history of cervical/arm pain lasting >3 months, previous diagnosis of a peripheral neuropathy, history of systemic illness or autoimmune disease, non–medically controlled hypertension and ongoing steroid treatment, and a history of whiplash injury or whiplash-related symptoms within the past 12 months. Participants were also assessed for their suitability to undergo MRI.

Proportionally age- and sex-matched healthy control (HC) participants were recruited through advertisements in university newsletters, social media, and posters in hospital waiting rooms. Participants were sent a participant information sheet, and then screened for suitability by telephone. Written informed consent was obtained by a member of the study team at the baseline appointment. In addition to the whiplash participants' exclusion criteria, HC participants were excluded if there was any history of whiplash injury, or they had received or were seeking treatment for neck, thoracic spine, or upper limb pain within the past 3 months.

### 2.4. Questionnaires

Whiplash-associated disorder grade II participants were asked to complete the neck disability index, the pain catastrophising scale (PCS), the Depression, Anxiety and Stress subscale and the posttraumatic stress disorder inventory-8 (PTSD-8). Neck disability index consists of 10 questions, each measured on a 6-point scale from 0 (no disability) to 5 (full disability), resulting in a maximum score of 50.^61,62^ The PCS consists of 13 questions scored from 0 (not at all) to 4 (all the time), resulting in a maximum score of 52 (30/52 being indicative of significant worrying related to pain), which can be further subdivided into rumination, magnification, and helplessness.^57^ The Depression, Anxiety and Stress subscale is a 42-item questionnaire that measures emotional states of depression, anxiety, and stress.^34^ The scale gives a total score of 126, with separate scores for each subscale between 0 and 42. A mild score is <6, moderate score 7 to 12, and severe ≥13. The PTSD-8 consists of 8 questions relating to how much symptoms have bothered the person since the traumatic event, scored from 0 (not at all) to 3 (most of the time), which can be subdivided into intrusion (from 4 items), avoidance, and hypervigilance (2 items each).^26^

### 2.5. Clinical assessment

All participants underwent a clinical assessment. The clinical assessments were performed by C.R. and J.F., who are physiotherapists, each with more than 10 years of clinical experience and postgraduate qualifications, as well as S.K., who was extensively trained by J.F. Demographic information (age, sex, height, weight) were collected, and a body chart and visual analogue scale for whiplash-related symptoms (0-100 mm) were completed to describe distribution, severity of current symptoms, and any subjective sensory changes. Because the researchers obtained the information from the participants on the initial appointment, it was not possible to blind them to the participants' status.

A neurological assessment of cutaneous hypersensitivity (sensory gain) and hypoaesthesia (sensory loss) of the upper extremities was performed using Neurotips and cotton wool to cover C5-T1 dermatomes. Isometric manual muscle strength and deep tendon reflexes of the upper limb were also assessed. Any participants with a neurological loss of function in 2 or more parameters within the same innervation territory (eg, loss of both strength and reflexes in C6) were excluded from the study, as they were considered to have frank nerve injury and as such would fit the inclusion criterion of WAD III, not WAD II.^53^

Tests for HNM of the brachial plexus, median, and ulnar nerves were performed bilaterally. The upper limb neurodynamic test-1 (ULNT1; median nerve bias) and ULNT3 (ulnar nerve bias) were both performed as previously described.^38^ For the ULNT1, the upper limb was positioned in 90° abduction and lateral rotation of the shoulder, elbow supination, wrist, and finger extension followed by elbow extension. For the ULNT3, the upper limb was positioned in 90° shoulder abduction, forearm pronation, wrist, and finger extension followed by elbow flexion. The ULNT1 and ULNT3 tests were considered “positive” when there was at least partial reproduction of a participant's symptoms, and these changed with structural differentiation.^38^ In addition, degree of elbow extension at the point of symptom onset was measured for the ULNT1 using a digital inclinometer (Trend; DLB, Swansea, United Kingdom).

Neural mechanosensitivity to pressure over the median and ulnar nerve trunks was established using an algometer (tip size = 1 cm^2^, Wagner, Greenwich, CT) and by applying digital pressure to the brachial plexus. The algometer was positioned over the ulnar nerve at the proximal cubital tunnel (CubT) and the carpal tunnel (CT) bilaterally with pressure applied at a rate of approximately 1 kg/second. Participants were asked to inform the researcher at the point where the pressure applied over the nerve changed to the first onset of pain. Where no pain was reported, pressure was stopped at 10 kg/cm^2^. Measurements were taken 3 times, and mean values were obtained for each site. The brachial plexus in the supraclavicular area was palpated digitally and graded according to symptom response: 0 = no pain or discomfort, 1 = local discomfort, 2 = local painful response, and 3 = referred pain/symptoms. Scores of 2 and 3 were considered a positive painful response to digital palpation. Phalen (applied passively for 60 seconds) and Tinel tests were performed bilaterally over the median nerve at the wrist.^32^ Briefly, Phalen test was performed by placing the participants' wrist joint into full flexion and sustaining this position. Tinel sign was performed by tapping over the median nerve at the wrist.

Heightened nerve mechanosensitivity was given a composite score of 0 or 1 based on a “positive test” from at least one of 3 tests: ULNT1, ULNT3, brachial plexus pain on palpation. Participants were grouped into positive (1) or negative HNM (0) based on this score.

### 2.6. Quantitative sensory testing

Quantitative sensory testing was performed according to the German Network for Neuropathic Pain protocol^46^ over the ventral aspect of the proximal phalanx of the index finger, where neuronal loss of function has previously been identified in people with WADII.^20^ Thermal detection and pain thresholds were measured using a Thermotester (Somedic AB, Norra Mellby, Sweden). Three repetitions were performed for cold detection threshold (CDT) and warm detection threshold (WDT). Thermal sensory limen (TSL) was obtained by alternating CDT and WDT in triplicate. The number of paradoxical heat sensations was determined during the TSL procedure. Cold pain threshold (CPT) and heat pain threshold (HPT) were then taken with 3 repetitions for each. Thermal measures were obtained using ramped stimuli of 1°C/second, starting at 32°C. The mean of 3 temperatures for each test was obtained.

Mechanical detection threshold (MDT) was obtained using the geometric mean of 5 descending and ascending stimuli using von Frey filaments starting with 16 mN (Optihair 2; MRC Systems GmbH, Heidelberg, Germany). Mechanical pain threshold was examined using pin prick (PP) stimulators (MRC Systems GmbH) with a range of 8 up to 512 mN. Five descending and ascending stimuli were collected before calculating the geometric mean.

Mechanical pain sensitivity (MPS) was determined with the same set of PP stimulators randomly applied to the testing site. Participants were asked to score pain from 0 to 100. For dynamic mechanical allodynia, 3 light touch (LT) stimuli (cotton wool, a cotton bud, and a standardised soft brush; MRC Systems GmbH) were applied randomly alongside the PP stimuli and were scored in the same way (0-100). In total, a shortened protocol of 14 PP and 6 nonnoxious stimuli was applied.^40^ Mechanical pain sensitivity was expressed as the geometric mean of the PP stimuli and dynamic mechanical allodynia as the geometric mean of the LT stimuli.

Windup ratio was tested by first asking the participant to rate 1 PP stimulus (256 mN) out of 100, before repeating the same stimulus 10 times at a rate of 1/second and asking the participant to rate the average pain out of the 10 stimuli. The average of 3 sets of stimuli was recorded as the windup ratio (quotient repeated/single stimulus).

Vibration detection threshold (VDT) was assessed using a Rydel Seiffer tuning fork, which was placed over the palmar aspect of the metacarpophalangeal joint of the index finger. Pressure pain threshold (PPT) was taken over the thenar eminence using an algometer (Wagner) by asking the participants to inform the researcher when the pressure stimuli had changed from pressure to first onset of pain. Both VDT and PPT were taken in triplicate, and the means were used for analysis.

Quantitative sensory testing was performed in the most symptomatic side in WADII participants and nondominant hand in HC participants.

### 2.7. Magnetic resonance imaging

#### 2.7.1. Magnetic resonance imaging acquisition

Images of the roots of the brachial plexus and DRG were obtained at each research site using a 3-T scanner of the same manufacturer and model (Siemens Prisma; Siemens Medical Solutions, Erlangen, Germany) with a dedicated 64-channel head/neck coil. Participants were positioned supine, and coronal images were obtained using a 2D multislice T1-weighted (echo time [TE] = 8.7 ms; repetition time [TR] = 500 ms; voxel size = 0.9 × 0.9 mm; slice thickness = 3 mm; (field of view) FoV = 330 mm) and a T2-weighted short tau inversion recovery 3D SPACE sequence (TE = 166 ms; TR = 3500 ms; TI = 230 ms; voxel size = 0.8 × 0.8 × 0.8 mm; FoV = 205 mm). T1-weighted images were used for anatomical reference.

For the wrist, the most symptomatic side was imaged, or the nondominant side in HCs. Participants were positioned in a supine superman position with their wrist placed in the middle of the scanner. A flex coil was positioned at the level of the CT. Axial images were acquired using a T2-weighted short tau inversion recovery sequence (TE = 21 ms; TR = 6030 ms; TI= 220 ms; voxel size = 0.4 × 0.4; slice thickness = 2 mm; interslice spacing = 0.2 mm; FoV = 100 mm).

#### 2.7.2. Magnetic resonance imaging analysis

**Figure 1.**
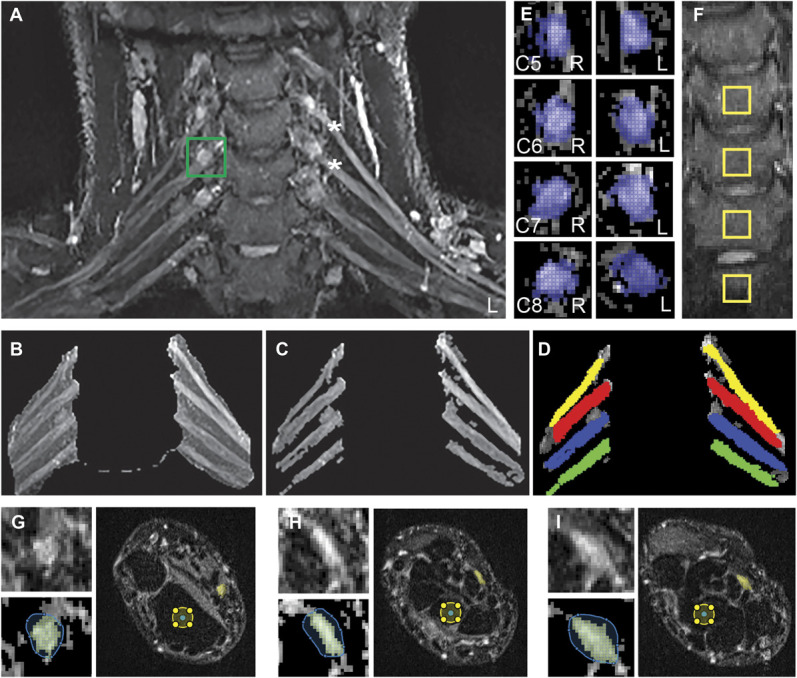
Image analysis of T2 weighted images of the brachial plexus, dorsal root ganglia (DRG), and median nerve at the wrist. (A) Example T2 maximum intensity projection image formed from extracted selected slices. A bounding box is shown positioned around the right C6 DRG (green square). (B) Freehand mask drawn around the roots of the plexus on the maximum intensity projection image. (C) Segmented regions overlaid to produce a complete segmented image of the roots of the brachial plexus. (D) Regions of interest overlaid on C5 to C8 roots. (E) Bounding boxes positioned around the C5-C8 DRG. (F) C5 to T1 vertebral bodies showing the 4 control ROIs (10 × 10 voxels). (G–I) Example axial slices of the median nerve at the wrist, at the (G) distal radioulnar joint, (H) proximal carpal row, and (I) distal carpal row. Upper left of (G–I): Enlarged image of the median nerve. Lower left of (G–I): Freehand mask drawn around nerve and segmented median nerve (yellow). Right of (G–I): Complete axial slice across wrist showing the segmented median nerve (yellow) and the control region of interest in the underlying bone (yellow circle). The T2-weighted scans were from WADII participants. Note the brighter C5 and C6 roots of the brachial plexus on the left side (*), which corresponded to the most symptomatic side in this participant. ROI, regions of interest; WADII, whiplash-associated disorder grade II.

Image files were initially coded and stored in 16-bit Digital Imaging and Communications in Medicine format. Digital Imaging and Communications in Medicine files were converted to NIfTI format and analysed by a blinded investigator using code developed in MATLAB (MathWorks, Natick, MA). For evaluation of the roots of the brachial plexus, T2-weighted coronal image slices with visible regions of the C5 to C8 roots were extracted (approximately 15 slices). A maximum intensity projection image was generated to enable visualisation of the root of the brachial plexus (Fig. 1A). A mask was created by drawing freehand around the roots of the plexus (Fig. 1B). This mask was consistently applied across all extracted slices using the same coordinates. Using thresholding and active contours model (also known as snakes), the roots of the plexus were segmented for each slice within the region of the mask. The segmented regions were overlaid to produce a complete segmented image of the roots of the brachial plexus (Fig. 1C). Overlapping voxels were averaged. From this image, regions of interest were drawn around the individual C5 to C8 roots (Fig. 1D), and median greyscale values were obtained.

Segmentation of the C5 to C8 DRG used similar methods. A bounding box was positioned around each visible DRG on a single image slice. Within the bounding box, the ganglion was segmented using thresholding and active contour modelling, and a median greyscale value was obtained within a manually drawn ROI (Fig. 1E).

To account for variations in position of individuals in the scanner, subsequent field inhomogeneities and, scanner differences at the 2 sites, the median T2 signal intensities of both the roots of the brachial plexus and DRG were normalised to regions of the C5-T1 vertebral bodies. The normalisation involved obtaining the median greyscale values for the C5 to T1 vertebral bodies from 4 regions of interest (10 × 10 voxels) within the centre of each vertebral body on 6 sequential coronal slices (total number of voxels = 2400; Fig. 1F). Scatter plots of the data confirmed a positive linear relationship between vertebral body (x-axis) and the roots of the brachial plexus, as well as the DRG, T2 signal (y-axis). Lines of best fit through the data did not intercept the origin (zero), and therefore, the average Y-intercepts were extrapolated to obtain constants (brachial plexus = 0.0012; DRG = 0.0015), which were subtracted from the T2 values of the roots of the brachial plexus and DRGs. T2 signal intensity ratios were determined by dividing these values by the median vertebral body T2 signal intensities.

For the analysis of the median nerve at the wrist, T2-weighted axial image slices were analysed at the level of the distal radioulnar joint, proximal and distal carpal row. At each site, a freehand mask was drawn around the median nerve on 3 adjacent slices. Thresholding and an active contours model were used to segment the nerve. Median greyscale values were obtained from the segmented slices, as well as median nerve area and aspect ratio. A circular control region of interest (radius = 10 voxels) was also positioned within the centre of a nearby bone (radius at the distal radio-ulnar joint, lunate at the proximal carpal row, and capitate at the distal carpal row) on each slice and the median greyscale value obtained for each region (Figs. 1G–I). Similar to the normalisation methods for both the roots of the brachial plexus and DRG, constants equivalent to the average Y-intercept for the median nerve T2 signal at each location (distal radioulnar = 0.00165; proximal carpal row = 0.00215; distal carpal row = 0.0024) were subtracted from individual T2 values, before dividing by the T2 signal intensity of the respective bone at each region.

### 2.8. Venepuncture and analysis of serum inflammatory cytokines

Whole blood was collected in Vacutainer tubes (BD Vacutainer SST II advanced tubes, FIsher Scientific, Loughborough, United Kingdom) from the antecubital fossa, and serum was extracted by centrifugation at 3000 rpm for 10 minutes at 4°C and stored at −80°C in cryotubes until processing.

Serum inflammatory mediators were assayed using multiplex electrochemiluminescence (V-Plex Proinflammatory Panel 1 (human) kit; Meso Scale Discovery, Rockville, MD). The mediators assayed were interferon-γ (IFN-γ), interleukin (IL) -1β, 2, 4, 6, 8, 10, 12p70, and 13, and tumour necrosis factor-α. All assays were performed according to the manufacturer's instructions. Samples were diluted 2-fold and assayed in duplicate. Plates were read using a Meso QuickPlex SQ (Meso Scale Discovery), and concentrations were determined from standard curves on each plate. Serum from WADII and HC participants was randomly assigned wells on the plates to control for batch effects.

### 2.9. Neuropathic Pain Special Interest Group classification of neuropathic pain

The certainty of neuropathic pain was classified for WADII participants as unlikely, possible or probable using the hierarchical classification system of the (international association for the study of pain) IASP Neuropathic Pain Special Interest Group.^19^ The classification of possible neuropathic pain is met when participants' symptoms are within a neuroanatomically plausible distribution with a medical history suggestive of a neurological lesion (eg, neuropathic pain descriptors such as burning, shooting, tingling). Probable neuropathic pain requires the aforementioned criteria, in addition to reduced sensory signs or allodynia in the same neuroanatomically plausible distribution. For the purposes of this study, we used the participants' description of symptoms and altered sensory examination findings in the main pain area. A diagnostic test would need to be undertaken to confirm the presence of definite neuropathic pain, and because the MRI scans were not clinically evaluated, this was not possible.

### 2.10. Data analysis

Sample size to ascertain differences in MRI T2 signal intensity between HCs and participants after whiplash was based on data from a chronic whiplash cohort,^23^ and full justification can be found in our previously published protocol.^44^ Ninety-six WADII participants and 32 HCs would be needed to detect an effect estimate of 0.06 for the mean nerve signal intensity difference (80% power, 5% significance).

Data were pseudonymised by assigning each participant a unique study-specific code and stored on a secure web platform (REDCap; Vanderbilt University, Nashville, TN). All MRI and blood marker analyses were blinded to participant group. Data were exported to GraphPad Prism (10.03) for analysis. The distribution of the data was checked for normality using the Kolmogorov–Smirnov test, and parametric or nonparametric methods were used as appropriate.

Quantitative sensory testing data were log transformed (except for CPT, HPT and VDT) to achieve normality and were expressed as *z*-scores, using the data of healthy participants age matched for the measurement site, with at least n = 7 HCs per decade.^4^ A positive *z*-score represents a gain of function, whereas negative *z*-scores indicates a loss of function.^46^ Mechanical pain sensitivity values included the addition of a constant 0.1 to avoid losing zero-rated values. For VDT *z*-scores, a ceiling effect was observed for the 20-year age decade due to a lack of variance, which prevented *z*-score calculation. Therefore, VDT *z*-scores for 20 and 30 year olds were derived from a combined total mean and standard deviation, incorporating both decades into one calculation. The remaining VDT *z*-scores were calculated for each decade.

For the T2 MRI analysis, data from each side were pooled in the HC group because there was no observed difference in intensity between the sides (*P* > 0.05). For the WADII participants, comparisons in T2 signal ratios were made between symptomatic side and pooled side for HCs and between symptomatic and less symptomatic sides.

Comparisons of mean (QST parameters) and medians (elbow ROM during ULNT1, CT and CubT PPT, T2 signal ratios, inflammatory mediator levels) between WADII and HC groups, and positive and negative HNM groups, were made using unpaired *t*-tests and Mann–Whitney tests, respectively. For comparisons of medians between participant's more and less symptomatic sides, Wilcoxon tests were used. For the median nerve, the mean T2 signal ratios at each level were compared using 2-way analysis of variance (location and group the independent variables) as data were normally distributed. For median nerve aspect ratios and area, Mann–Whitney tests were used to compare between the groups at each location. Multiple proportions were compared using Fisher exact tests. Spearman ranks correlations were used to investigate the relationship of T2 signal ratios with clinical tests and demographic data. We accepted *r* values of ≥0.4 as a measure of a moderate correlation.^50^ Bonferroni corrections were used to adjust separate analyses for multiple testing for T2 signal ratios of the brachial plexus and DRG, and blood serum inflammatory mediator analyses (corrected α provided for each result). The QST data were not corrected for multiple comparisons because correcting for this number of measures (n = 11), many of which are mechanistically unrelated may lead to an underestimation of any real effect.^47^

### 2.11. Standardisation across sites

All clinical test procedures were piloted between sites to ensure standardisation. Interrater reliability of elbow ROM using the inclinometer for nerve mechanosensitivity testing was performed. Intraclass correlation coefficient was 0.873 (95% confidence interval [CI]: 0.64-0.96, *P* < 0.0001), standard error of measurement was 5.14°, and smallest detectable difference was 14.25°. The German Network of Neuropathic Pain QST protocol has been shown to have substantial interrater and intercentre reliability.^21,35^ The same make and models of MRI scanners and coils were used at both sites.

## 3. Results

### 3.1. Participant demographics

Participant demographics are summarised in Table 1. One hundred twenty-two WADII participants and 43 HCs were recruited. Of these, 102 WADII participants and 37 HCs underwent MRI scans. Groups were comparable for age, height, and weight. All HCs had normal muscle strength and reflexes on neurological testing. The median score for neck disability was 15, indicating a moderate disability. Participants had low levels of pain-related worrying as indicated with a median overall score of 13 on the PCS. Although participants had mild symptoms of depression and anxiety, they had moderate symptoms of stress, which coincided with all 3 dimensions of the PTSD-8 questionnaires having median scores greater than 3, indicating high levels of posttraumatic stress in this cohort.^1^

**Table 1:** Participant demographics.

	Whiplash	Healthy control
No. of participants	122	43
Age (mean, SD), y	36.6 (12.2)	35.9 (11.6)
Sex (n = females) (%)	69 (57%)	22 (51%)
Height (median, IQR) (n = 120 WADII, n = 42 HC)	170 (14.6)	173.9 (18.2)
Weight (median, IQR) (n = 120 WADII, n = 42 HC)	76.5 (22.4)	74.5 (27.93)
VAS (mean, SD) (n = 121)	39.8 (21.8)	N/A
Collision direction (forward, side, rear) % (n)	27% (33), 37% (45), 36% (44)	N/A
Time since injury (mean days [SD])	23.7 (7.4)	N/A
Symptomatic side (right), % (n)	57% (70)	N/A
Proportion with bilateral symptoms, % (n)	49% (60)	N/A
Proportion with self-reported sensory changes, % (n)	42% (51)	N/A
Neck disability index (median, IQR) (n = 119)	15 (10)	N/A
Pain catastrophising scale (median, IQR) (n = 109)	13 (15)	N/A
DASS score (median, IQR)		
Depression	5.5 (11)	N/A
Anxiety	6 (9)	
Stress (n = 116)	12 (12)	
PTSD-8: (median, IQR)		
Intrusion	9.5 (4)	N/A
Avoidance	4 (3)	
Hypervigilance (n = 114)	5 (3)	
Positive Tinel sign % (n) (n = 118 WADII, n = 39 HC)	39% (46)	6.98% (9)
Positive Phalen test % (n) (n = 113 WADII, n = 39 HC)	36% (41)	4.7% (6)
Positive ULNT1% (n) (n = 116 WADII, n = 37 HC)	35% (41)	0%
Positive ULNT3% (n) (n = 115 WADII, n = 37 HC)	17% (20)	0%
Neuropathic pain (NeuPSIG classification)		
Probable	36% (44)	N/A
Possible	27% (33)	
Unlikely % (n) (n = 122)	37% (45)	

DASS, Depression Anxiety Stress Scale; HC, healthy control; IQR, interquartile range; PTSD-8, posttraumatic stress disorder inventory-8; ULNT1, upper limb neurodynamic test 1 (median nerve biased); ULNT3, upper limb neurodynamic test 3 (ulnar nerve biased); WADII, whiplash-associated disorder grade II; VAS, visual analogue scale.

### 3.2. Symptom distribution

**Figure 2.**
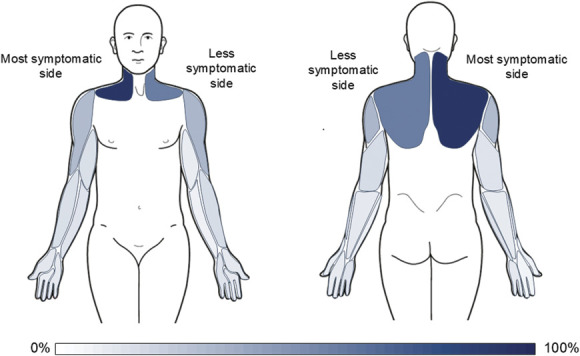
Symptom distribution in WADII participants. Percent of participants reporting symptoms is indicated in blue, with darker colours indicating higher percentages. WADII, whiplash-associated disorder grade II.

The distribution of symptoms in WADII participants is shown in Figure 2. The most common region for symptoms was the cervical spine on the most symptomatic side (92%). Symptoms in the shoulder, arm, forearm, or hand were reported in 65% of participants.

### 3.3. Magnetic resonance imaging measures of peripheral neuroinflammation

#### 3.3.1. Brachial plexus

**Figure 3.**
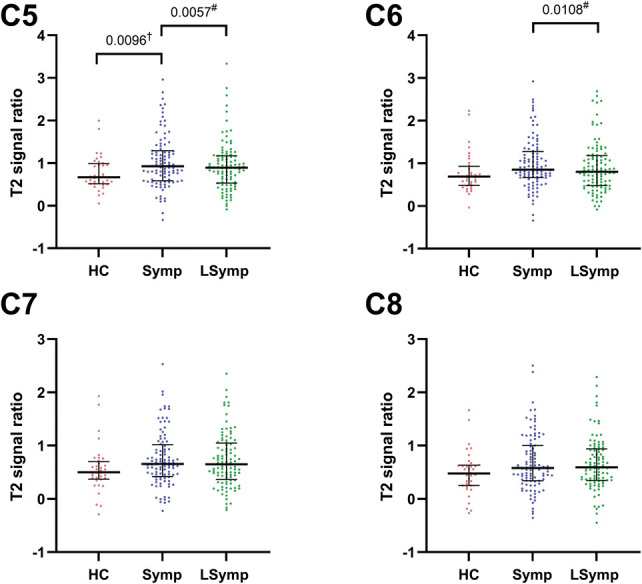
T2-signal ratios for the roots of the brachial plexus. Pooled data from the healthy control (HC) group is compared with symptomatic side of WADII participants (†Mann–Whitney tests), Symptomatic (Symp) and less symptomatic (LSymp) sides are also compared (#Wilcoxon signed-rank test). The median values are shown (horizontal bars). Upper and lower horizontal bars represent IQRs. α = 0.0125. IQR, interquartile range; WADII, whiplash-associated disorder grade II.

For the roots of the brachial plexus, the T2 signal ratio on the symptomatic side in WADII participants was significantly higher compared with HCs for C5 (*P* = 0.0096), but not for C6 (*P* = 0.0682), C7 (*P* = 0.0415), or C8 (*P* = 0.0644; α = 0.0125). For both C5 and C6 roots, T2 signal ratios were significantly increased on the symptomatic side compared with the less symptomatic side for WADII participants (*P* = 0.0057 and 0.0108 for C5 and C6 nerve roots, respectively; α = 0.0125; Fig. 3; refer to Fig. 1A for example image), although no differences were found between sides for C7 (*P* = 0.3067) or C8 roots (*P* = 0.5095). Supplementary table 1 displays complete data (http://links.lww.com/PAIN/C234).

#### 3.3.2. Dorsal root ganglia

**Figure 4.**
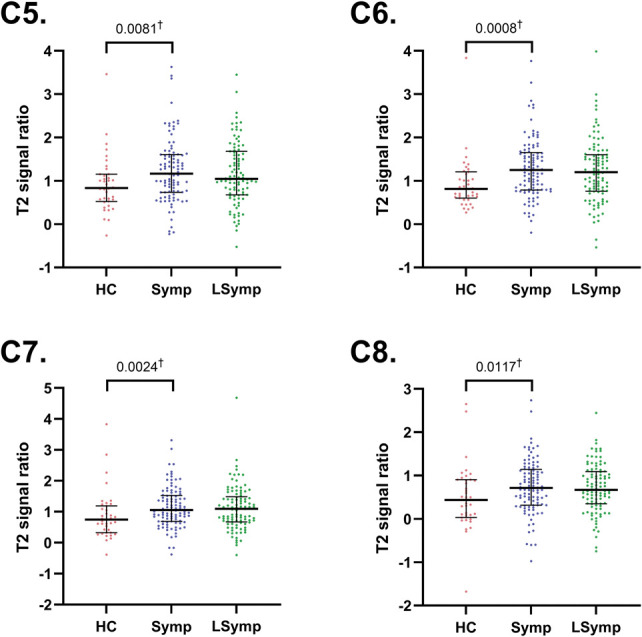
T2 signal ratios for the C5-C8 dorsal root ganglia. Pooled data from the healthy control (HC) group is compared with symptomatic side of WADII participants (†Mann–Whitney tests), Symptomatic (Symp) and less symptomatic (LSymp) sides are also compared. The median values are shown (horizontal bars). Upper and lower horizontal bars represent IQRs. α = 0.0125. IQR, interquartile range; WADII, whiplash-associated disorder grade II.

For the DRGs, the T2 signal ratio on the symptomatic side in WADII participants was significantly higher compared with HCs for C5 (*P* = 0.0081), C6 (*P* = 0.0008), C7 (*P* = 0.0024), and C8 (*P* = 0.0117; α = 0.0125; Fig. 4). There were no observed differences between symptomatic and less symptomatic sides (*P* > 0.0670). Supplementary table 1 displays complete data (http://links.lww.com/PAIN/C234).

#### 3.3.3. Median nerve

**Figure 5.**
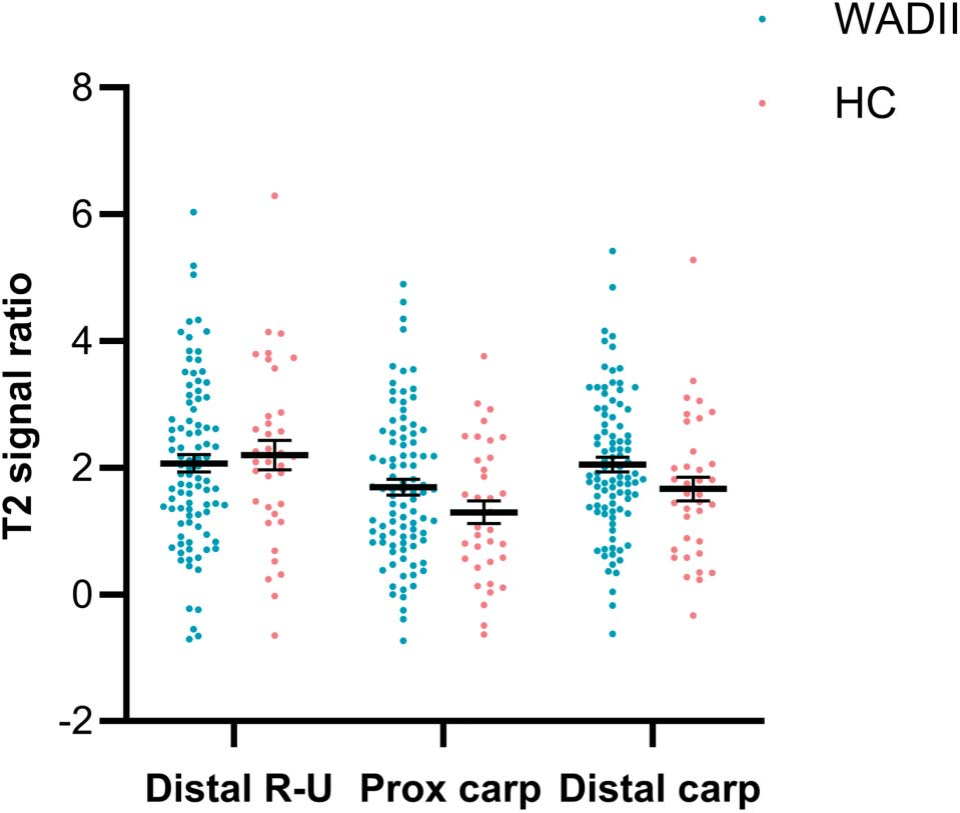
T2 signal ratios for the median nerve at the wrist of the most symptomatic side at 3 levels: Distal R-U, distal radioulnar joint; Prox carp, proximal carpal row; Distal carp, distal carpal row. HC, healthy control.

There was a significant interaction between location and group (*P* = 0.0167), with significant differences between T2 signal ratio at each location in both the WADII and HC groups (*P* > 0.0462). However, there were no significant differences in median nerve T2 signal ratio between WADII participants and HCs at any wrist location (*P* > 0.0709; Fig. 5). There were no significant differences in median nerve area or aspect ratio at the wrist between the groups (Table 2). Supplementary table 1 displays complete data (http://links.lww.com/PAIN/C234).

**Table 2:** Median nerve parameters at the wrist.

Measure	Location	WADII (median, IQR)	Healthy control (median, IQR)	*P*
Area	Distal R-U	68.17 (26.42)	62.67 (29.67)	0.1185
	Prox carp	81.17 (23.75)	77.83 (22.59)	0.4515
	Distal carp	73.17 (23.91)	72.17 (19.66)	0.3149
Aspect ratio	Distal R-U	1.26 (0.19)	1.29 (0.19)	0.7658
	Prox carp	1.67 (0.46)	1.71 (0.35)	0.6691
	Distal carp	1.77 (0.51)	1.86 (0.25)	0.1898

Distal R-U, distal radio-ulnar joint; Distal carp, distal carpal row; IQR, interquartile range; Prox carp, proximal carpal row; WADII, whiplash-associated disorder grade II.

### 3.4. Presence of heightened nerve mechanosensitivity

#### 3.4.1. Upper limb neurodynamic tests

**Figure 6.**
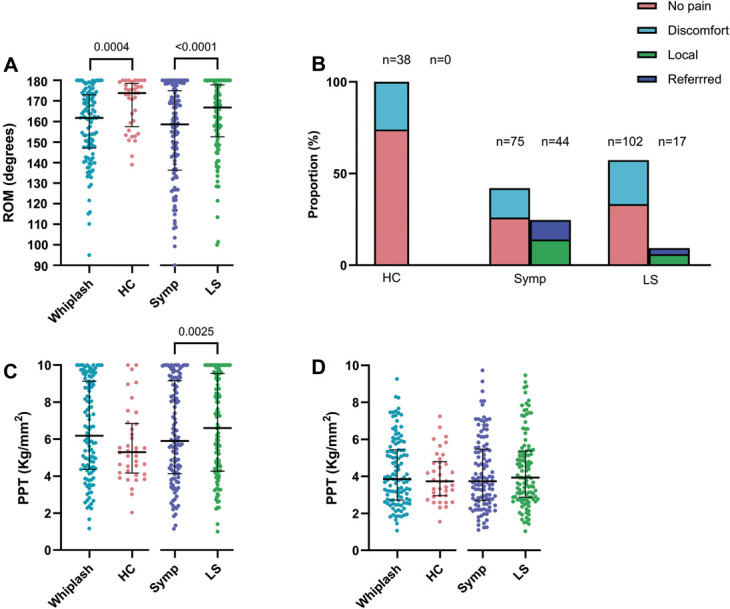
Heightened nerve mechanosensitivity measures. (A) Elbow extension range of motion during the ULNT1 (median nerve bias). (B) Digital palpation of the brachial plexus. The proportion of participants with no pain, discomfort, and local and referred pain is shown. (C) Pressure pain thresholds over the median nerve at the carpal tunnel. (D) Pressure pain thresholds over the ulnar nerve at the cubital tunnel. In (A, C, and D), pooled data from both sides are compared (whiplash and HC groups), as well as data from the symptomatic and less symptomatic sides; the medians are shown (horizontal bars). Upper and lower error bars represent the IQRs. HC, healthy control; IQR, interquartile range; LSymp, less symptomatic side; ROM: range of motion; Symp, symptomatic side; ULNT1, upper limb neurodynamic test-1.

Thirty-five percent of WADII participants had a positive ULNT 1 (median nerve bias), and 17% had a positive ULNT3 (ulnar nerve bias) on their most symptomatic side. There was reduced elbow ROM during ULNT1 in WADII participants compared with HCs (*P* = 0.0004). Elbow ROM for WADII participants was also reduced on the most symptomatic side compared with the less symptomatic side (*P* < 0.0001; Fig. 6A).

#### 3.4.2. Pressure over upper limb nerves

A greater proportion of WADII participants reported referred or localised pain on their symptomatic side (37%) compared with the less symptomatic side (14%) and HCs (0%) during digital palpation over the trunks of the brachial plexus (*P* <0.0001; Fig. 6B).

There were no significant differences in PPT (*P* = 0.1238) over the median nerve at the CT in WADII participants compared with HCs (sides pooled), although there was a significant reduction in PPT on the more symptomatic compared with the less symptomatic side (*P* = 0.0025; Fig. 6C). There were no significant differences in PPT over the ulnar nerve at the CubT in WADII participants compared with HCs (*P* = 0.5823) or between symptomatic and less symptomatic sides in WADII (*P* = 0.2193; Fig. 6D). Supplementary table 1 displays complete data (http://links.lww.com/PAIN/C234).

### 3.5. Serum inflammatory mediators

**Figure 7.**
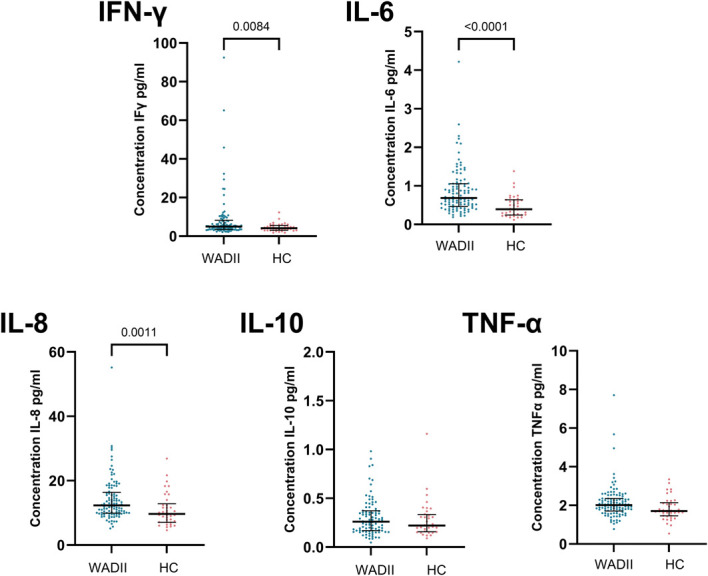
Blood serum concentrations of inflammatory mediators. Values for interferon-γ (IFN-γ) and IL-10 have been cropped to 100 and 2 pg/mL for display (missing values IFN-γ n = 2, 108.6, 138.89; IL-10 n = 1, 5.74, α = 0.01). HC, healthy control; IL, interleukin; WADII, whiplash-associated disorder grade II.

Five of the 10 inflammatory mediators (IL-1β, IL-2, IL-4, IL-12, and IL-13) were below detectable levels in most participants and were therefore not analysed further. There were significantly higher levels of IFN-γ, IL-6, and IL-8 in WADII participants compared with HCs (*P* = 0.0084, *P* < 0.0001, and *P* = 0.0011, respectively) but not IL-10 (*P* = 0.3917) or tumour necrosis factor-α (*P* = 0.0155; α = 0.01; Fig. 7). Supplementary table 1 displays complete data (http://links.lww.com/PAIN/C234).

### 3.6. Changes to cutaneous sensation

#### 3.6.1. Neurological assessment

**Figure 8.**
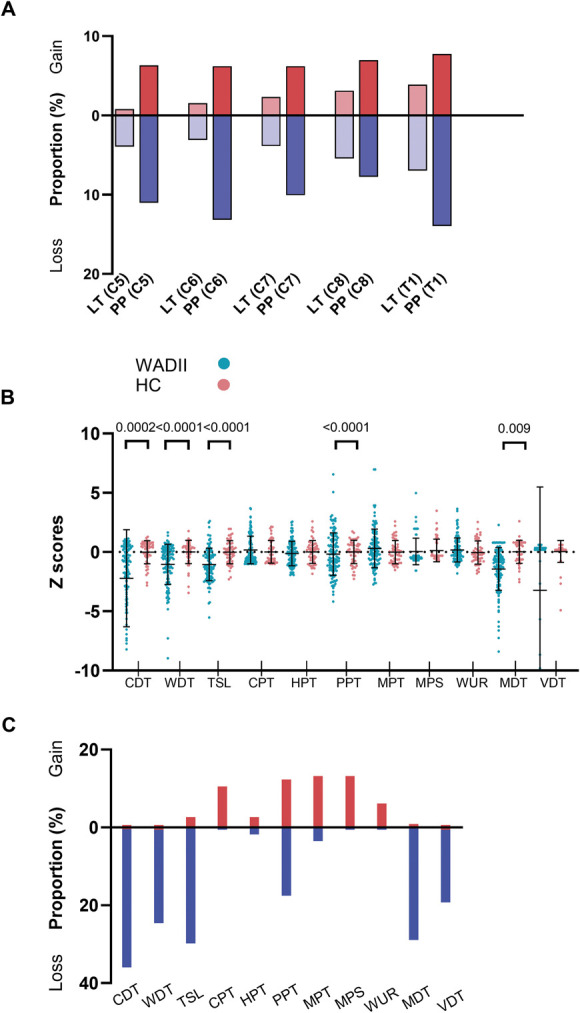
(A) Proportion of participants with sensory changes upon bedside neurological examination to light touch and pin prick in the upper limb dermatomes on the symptomatic side (C5-T1; n = 122). (B) Quantitative sensory testing profiles over the index finger in WADII participants and HCs. *Z*-scores cropped to −10 for display (missing values CDT n = 6, range −10.438 to −26.723, VDT n = 21, range −14.667 to −39.796). (C) Proportion of WADII participants with a gain or loss of sensory function based on QST measures (individuals with *Z*-scores ±1.96 SD of HCs). CDT, cold detection threshold; CPT, cold pain threshold; HC, healthy control; HPT, heat pain threshold; LT, light touch; MDT, mechanical detection threshold; MPT, mechanical pain threshold; MPS, mechanical pain sensitivity; PP, pin prick; PPT, pressure pain threshold; QST, quantitative sensory testing; TSL, thermal sensory limen; VDT, vibration detection threshold; WADII, whiplash-associated disorder grade II; WDT, warm detection threshold; WUR, windup ratio.

Figure 8A shows the proportion of WADII participants with a gain or loss of sensation in the C5-T1 dermatomes. Clinical signs of altered sensation (gain or loss) in one or more upper limb dermatomes were found on the most symptomatic side in 47% (n = 57/122) of WADII participants. Eighteen percent of WADII participants had a gain in sensation, compared with 37% who reported a loss of sensation. In 11 (9%) of these participants, both a gain and loss of sensation were found in different upper limb dermatomes.

#### 3.6.2. Quantitative sensory testing

Quantitative sensory testing profiles over the index finger for WADII participants and HCs are shown in Figure 8B. There were significant reductions in both thermal (CDT [*P* = 0.0002], WDT and TSL [*P* < 0.0001]) and mechanical (MDT [*P* < 0.0001] and VDT [*P* = 0.009]) sensory measures in participants following whiplash compared with HCs. The proportion of WADII participants with a gain and loss of function based on the QST measures are shown in Figure 8C. Supplementary table 1 displays complete data (http://links.lww.com/PAIN/C234).

### 3.7. Association between nerve T2 signal ratio and clinical measures

There was a significant positive correlation between pooled brachial plexus T2 signal ratios (max value from C5-C8) and age for the WADII participants (*P* < 0.0001, *r* = 0.48 [95% CI: 0.31-0.62], but not for HCs [*P* = 0.377]). A similar positive correlation was also observed between DRG T2 signal ratio (max value from C5-C8) and age for the WADII participants (*P* < 0.0001, *r* = 0.43 [95% CI: 0.24-0.58]). In contrast to the brachial plexus, DRG T2 signal ratio correlated negatively with age in the HC group (*P* = 0.0013, *r* = −0.4 [95% CI: −0.65 to 0.07]).

There were no other significant moderate correlations between T2 brachial plexus or DRG signal ratios and the other clinical measures, which included our surrogate measures of peripheral neuroinflammation (Supplementary table 2 and 3 displays full results, http://links.lww.com/PAIN/C234).

### 3.8. Comparison of clinical measures in people with and without heightened nerve mechanosensitivity

**Figure 9.**
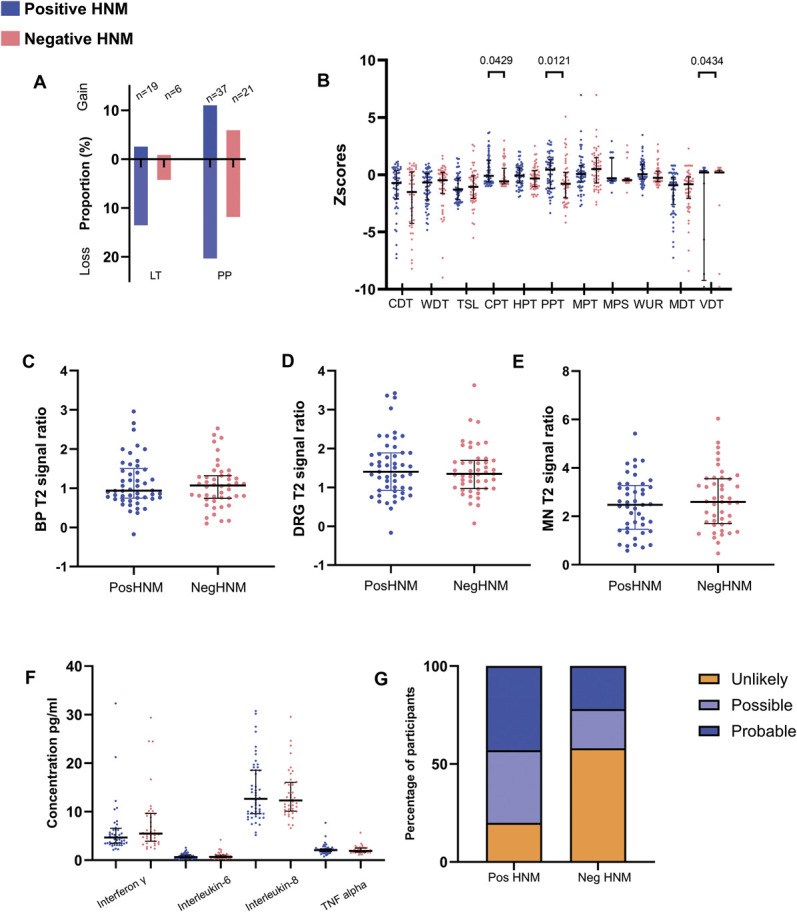
Comparison of clinical measures in WADII participants with and without heightened nerve mechanosensitivity. (A) Proportion of participants with sensory changes to light touch (LT) or pin prick (PP) in at least one upper limb dermatome (n = 118; participants with both a gain and loss of function in one of the dermatomes; LT n = 0, PP n = 7). (B) Quantitative sensory testing *z*-scores. *Z*-scores for VDT cropped to −10 for display (missing values n = 21, range −14.667 to −39.796). (C–E) MRI T2 signal ratios for (C) brachial plexus, (D) DRG, and (E) median nerve. Individual values represent the highest T2 value for the levels of the roots of the brachial plexus, DRG, and median nerve location. (F) Serum inflammatory mediator concentrations. (G) Proportion of participants with unlikely, possible and probable neuropathic pain according to the presence of heightened nerve mechanosensitivity (*P* = 0.0002; n with positive HNM = 65, n with negative HNM = 55). In (A–F), positive HNM = blue, negative HNM = red. CDT, cold detection threshold; CPT, cold pain threshold; DRG, dorsal root ganglia; HNM, heightened nerve mechanosensitivity; HPT, heat pain threshold; LT, light touch; MDT, mechanical detection threshold; MRI, magnetic resonance imaging; MPS, mechanical pain sensitivity; MPT, mechanical pain threshold; PP, pin prick; PPT, pressure pain threshold; TSL, thermal sensory limen; VDT, vibration detection threshold; WDT, warm detection threshold; WADII, whiplash-associated disorder grade II; WUR, windup ratio.

Clinical measures were compared between WADII participants with (positive) and without (negative) signs of HNM. The percentage of participants with HNM based on a composite score was 55% (65/118). Although there was an observed increase in the proportion of participants with a loss of LT in the positive compared with negative HNM groups, this difference was not significant (*P* > 0.999; Fig. 9A).

For QST, those with positive HNM had a greater gain in function in CPT (*P* = 0.0429) and PPT (*P* = 0.0121) and a greater loss in function of VDT (*P* = 0.0434) than those with negative HNM (Fig. 9B).

There were no differences in T2 signal ratios at any location (Figs. 9C–E) or levels of serum inflammatory mediators (Fig. 9F) between those with positive and negative HNM (*P* > 0.118).

Using the Neuropathic Pain Special Interest Group grading system to classify the likelihood of neuropathic pain, a greater proportion of participants with possible or probable neuropathic pain had HNM (*P* = 0.0002; Fig. 9G).

## 4. Discussion

The primary aim of this study was to identify evidence for the presence of peripheral neuroinflammation in WADII. We identified elevated MRI T2 signal ratios of the C5-8 DRGs and the C5 root of the brachial plexus in participants with WADII compared with healthy participants, consistent with neuroinflammation. Secondary outcomes consistent with neuroinflammation (ie, elevated serum inflammatory mediators, HNM, and somatosensory hyperalgesia in patients with WADII) support these findings. Our findings suggest that WADII is a complex disorder and that minor nerve injury and neuroinflammation may play a role in a subgroup of patients with WADII.

### 4.1. Evidence for peripheral neuroinflammation on magnetic resonance imaging

The increased T2 signal ratios from the roots of the brachial plexus and cervical DRG in WADII participants suggest the presence of intraneural oedema associated with neuroinflammation in a subgroup of patients^10^ and align to our previous findings in chronic WADII.^23^ The causes of peripheral neuroinflammation following whiplash are unclear, although it may be the result of a sudden increase in tension of the brachial plexus during the cervical acceleration–deceleration movement^28,51^ or compression of the spinal nerves/DRGs because of narrowing of the cervical intervertebral foramina.^29^ Although systemic oedema because of circulatory and metabolic diseases and inflammation associated with autoimmune diseases could increase T2 nerve signal, such conditions were part of the exclusion criteria. From the current data, it is not possible to elucidate whether any of the participants had inflammation before the injury. However, the strict inclusion criteria, and the inclusion of a HC group recruited with the same exclusion criteria, reduces this likelihood.

The increased T2 signal ratio at multiple levels of the brachial plexus (C5-C6) and DRG (C5-C8) suggests widespread neuroinflammation, which also corresponded to the presence of cutaneous sensory changes in multiple upper limb dermatomes. Although T2 signal was not associated with symptom severity, the elevated T2 signal ratios on the most symptomatic brachial plexus roots of C5 and C6 suggests that peripheral neuroinflammation preferentially occurs on the more affected side.

Unlike our previous findings in people with chronic WADII,^23^ we did not find an increase in T2 signal ratio of the median nerve at the wrist in those with acute WADII. It is possible that such distal changes only occur at later time points following whiplash injury.

The positive correlation between age and T2 signal ratios in WADII participants but not HCs suggests that age may predispose individuals to greater neuroinflammation after whiplash. For example, age-related changes to the viscoelastic properties of peripheral nerves,^17,24,60^ including the endoneural blood vessels, may increase their susceptibility to trauma. Furthermore, because aging is associated with an increase in inflammation,^33^ the observed positive correlation in WADII participants may be the effect of a greater inflammatory response with age.

### 4.2. The contribution of heightened nerve trunk mechanosensitivity

Heightened nerve mechanosensitivity was a significant feature of acute WADII, with more than half of the cohort showing signs. Because localised neuroinflammation in animal studies causes axonal mechanosensitivity in apparent uninjured nociceptors,^6,12,13,22^ the heightened nerve trunk mechanosensitivity reported in this study may be a consequence of similar neuroinflammation. Although T2 signal ratios and inflammatory mediator concentrations were not different between those with and without HNM, the increase in cold and pressure hyperalgesia and higher proportion of participants with possible or probable neuropathic pain in those with HNM may suggest mechanistic differences between the groups. However, the precise mechanism is unclear in this group.

In contrast to signs of somatosensory hyperalgesia, the observed loss of vibration detection in WADII participants in the positive HNM group suggests a loss of Aβ-fibre function. This finding indicates a more complex pathology in some WADII participants (see section 4.4). However, because there were no other significant reductions in mechanical detection in the HNM group, this significant finding may be because of several very low VDT *z*-scores potentially associated with a ceiling effect of the measure.

### 4.3. A potential role for proinflammatory cytokines

The increase in serum levels of IFN-γ, IL-6, and IL-8 in WADII participants is consistent with systemically detectable inflammation following whiplash injury. Several studies have also shown a similar increase in inflammatory markers following whiplash.^31,56^ There is substantial evidence to suggest a role for cytokines in neuropathic pain.^16^ Our study cannot ascertain whether the increase in inflammatory mediators in WADII participants is a direct result of a peripheral nerve injury, as acutely elevated cytokines may arise for numerous direct and indirect mechanisms such as other soft tissue injury and even PTSD,^41^ which was present in some of the cohort.

### 4.4. Complex somatosensory phenotypes in whiplash-associated disorder grade II

Both the neurological assessment and QST confirmed the presence of somatosensory hyperalgesia in a proportion (18%) of people with WADII, which is consistent with the reports of mechanical and thermal evoked cutaneous hypersensitivities in animal models of peripheral neuroinflammation.^6,15,42,48^ However, there was notable variation between individuals, with a loss of function being more frequently observed (37%). Such variation may reflect the heterogeneity of WADII and the presence of multiple phenotypes, which would explain the lack of correlation between elevated T2 signal ratios and clinical measures. This finding suggests potential differences in the severity of the underlying pathology because a loss of function is typically associated with structural changes to axons and/or their myelin sheaths. Indeed, peripheral neuroinflammation can cause demyelination and axonal damage.^9,58^ Alternatively, inflammation around intact axons may also contribute to loss of function. For example, in the neuritis model, inflammation disrupts axonal transport along apparent uninjured axons.^11,14^ Therefore, a loss of cutaneous function could result from the reduced transport of sensory transducers to the peripheral terminals of intact axons. The presence of a frank nerve injury leading to extensive axonal degeneration can be ruled out because of the strict exclusion criteria.

### 4.5. Association between measures of peripheral neuroinflammation

The lack of correlation between T2 signal ratio and measures of HNM or QST parameters was unexpected, especially because we hypothesised that both HNM and somatosensory hyperalgesia are surrogate measures of neuroinflammation. The reason for these discrepancies is multifaceted but may in part be related to the sensitivity of our tests and because an increased T2 signal is not a marker of a specific inflammatory event. Accordingly, in animal models of neuritis, the time course of axonal mechanical sensitivity and pedal hypersensitivities does not correlate with the presence of peripheral neuroinflammation, which persists beyond the resolution of these changes.^7,12,48,49^ Therefore, the neural changes that lead to HNM and somatosensory hyperalgesia may be driven by a particular component of inflammation, rather than simply the presence of inflammation. The lack of correlation between T2 signal ratio and cytokine levels is most likely due to the presence of general inflammation caused by the injury.

### 4.6. Clinical implications

The findings from this study indicate that a subgroup of individuals with WADII have a minor peripheral nerve injury consistent with a neuroinflammatory phenotype. Our findings from specialised assessments are in direct contradiction with the Quebec whiplash criteria for WADII, which are based on traditional bedside testing only. Our findings imply that the whiplash diagnostic grades may need revising, for instance, by incorporating assessments for sensory changes that are consistent with peripheral neuroinflammation, including tests for HNM as a subgroup of WADII.

The current management of individuals with WADII involves treatments for nociceptive pain, such as simple analgesics (paracetamol and (non steroidal antiinflammatory drugs) NSAIDs) and using a system, such as the (World Health Organisation) WHO ladder, to appropriately escalate medication where required.^27^ Additionally, exercise and joint mobilisations for pain relief and behavioural management strategies may be most appropriate for managing people with WADII,^37,43^ albeit with overall small effects. The presence of a minor peripheral nerve injury, such as neuroinflammation, and symptoms that are typically considered neuropathic render a different management approach. This might involve pharmacological treatments for neuropathic pain such as antidepressants or anticonvulsants,^3,36,39^ as well as novel therapies to treat neuroinflammation,^8^ and other conservative management strategies such as neurodynamic interventions.^45^ As such, it is imperative that a comprehensive assessment is made of individuals following a whiplash injury, including detailed neurological screening and tests for HNM.

## 5. Conclusions

This study demonstrates evidence for the presence of peripheral neuroinflammation in some individuals with acute WADII. Our primary measure shows a significant increase in T2-weighted signal ratios of some roots of the brachial plexus and multilevel DRGs, consistent with peripheral neuroinflammation. Secondary measures including raised levels of inflammatory mediators, somatosensory hyperalgesia, and HNM further support the findings. Clinicians should be mindful of the potential for nerve involvement in some patients presenting as WADII and ensure a comprehensive examination of the nervous system and tests for HNM are performed.

## Conflict of interest statement

The authors have no conflicts of interest to declare.

## Supplemental digital content

Supplemental digital content associated with this article can be found online at http://links.lww.com/PAIN/C234.

## Supplementary Material

SUPPLEMENTARY MATERIAL
