# Supplementary material for: Evidence for peripheral neuroinflammation after acute whiplash
Source: Pain. 2025 Mar 4;166(10):2285–99. doi: 10.1097/j.pain.0000000000003560 (PMC12444895; doi:10.1097/j.pain.0000000000003560)
Supplement: SUPPLEMENTARY MATERIAL [file jop-166-2285-s002.pdf]

Supplementary table 1

Measure	WADII (Symp)	WADII (LSymp)	Healthy control	p value (HC V WADII)
T2BPC5 Median (IQR)†	0.93 (0.71)	0.89 (0.64)	0.67 (0.47)	0.0096*
T2BPC6 Median (IQR)†	0.85 (0.61)	0.80 (0.70)	0.69 (0.45)	0.0682
T2BPC7 Median (IQR)†	0.66 (0.60)	0.65 (0.68)	0.50 (0.33)	0.0415
T2BPC8 Median (IQR)†	0.58 (0.66)	0.59 (0.59)	0.48 (0.38)	0.0644
T2DRGC5 Median (IQR)†	1.17 (0.87)	1.05 (1.00)	0.83 (0.63)	0.0081*
T2DRGC6 Median (IQR)†	1.25 (0.86)	1.2 (0.84)	0.81 (0.61)	0.0008*
T2DRGC7 Median (IQR)†	1.06 (0.84)	1.09 (0.81)	0.74 (0.87)	0.0024*
T2DRGC8 Median (IQR)†	0.71 (0.82)	0.67 (0.74)	0.43 (0.87)	0.0117*
T2 median nerve distal r-u joint Mean (SE)†	2.07 (0.14)	n/a	2.2 (0.23)	0.6385
T2 median nerve distal carpal row Mean (SE)†	1.69 (0.12)	n/a	1.3 (0.18)	0.0709
T2 median nerve proximal carpal row Mean (SE)†	2.05 (0.11)	n/a	1.67 (0.19)	0.0848
Elbow extension ROM ULNT1 Median (IQR)†	68.6 (38.62)	76.8 (25.27)	83.8 (21.07)	0.0004*
PPT Cubital tunnel Median (IQR)†	3.73 (2.77)	3.93 (2.5)	3.73 (1.85)	0.219
IFN-γ Median (IQR)†	4.99 (4.72)	n/a	4.16 (2.56)	0.0084*
IL-6 Median (IQR) †	0.68 (0.59)	n/a	0.39 (0.39)	0.0001*
IL-8 Median (IQR) †	12.33 (6.44)	n/a	9.67 (5.71)	0.0011*
IL-10 Median (IQR)†	0.26 (0.20)	n/a	0.22 (0.18)	0.39
TNF-α Median (IQR)†	2.01 (0.65)	n/a	1.71 (0.68)	0.0155
CDT Mean (SE)	-2.21 (0.37)	n/a	-0.01 (0.14)	0.0002*
WDT Mean (SE)	-1.04 (0.15)	n/a	0.00 (0.14)	<0.0001*
TSL	-1.05 (0.12)	n/a	-0.01 (0.14)	<0.0001*
CPT Mean (SE)†	0.17 (0.11)	n/a	0.02 (0.13)	0.324
HPT Mean (SE)†	-0.13 (0.09)	n/a	0.01 (0.14)	0.42
MDT Mean (SE)	-1.43 (0.16)	n/a	0.02 (0.14)	<0.0001*

MPT Mean (SE) †	0.31 (0.15)	n/a	-0.01 (0.14)	0.1999
MPS Mean (SE) †	0.05 (0.10)	n/a	0.13 (0.14)	0.6435
WUR Mean (SE) †	0.18 (0.09)	n/a	-0.04 (0.14)	0.2012
VDT Mean (SE)	-3.23 (0.80)	n/a	0.05 (0.13)	0.009*
PPT Mean (SE) †	-0.18 (0.16)	n/a	0.01 (0.14)	0.49

Median (IQR) or mean (SE) data with p values for unpaired comparisons. Symp, symptomatic side; LSymp, less symptomatic side; IQR, interquartile range; SE, standard error of mean; BP, roots of brachial plexus; DRG, dorsal root ganglia; distal R-U, distal radio-ulnar joint; ROM, range of motion, ULNT1, upper limb neurodynamic test median nerve bias; PPT, pressure pain threshold; CDT, cold detection threshold; WDT, warm detection threshold, TSL, thermal sensory limen; CPT, cold pain threshold; HPT, heat pain threshold; MDT, mechanical detection threshold; MPT, mechanical pain threshold; MPS, mechanical pain sensitivity; WUR, wind up ratio; VDT, vibration detection threshold * is statistically significant (α varies from $p<0.05$ to $p<0.01$ dependent on analysis), † surrogate measures of neuroinflammation.

Supplementary table 2

Variable 1	Variable 2	p value	r value (Spearman ranks) (95% CI)
T2 BP Signal ratio whiplash (max) †	Age	<0.0001	0.48 (0.31-0.62)
	Height	0.14	-0.15 (0.34-0.05)
	Weight	0.006	0.28 (0.08-0.46)
	QST z scores: CDT	0.6	-0.03 (-0.24-0.18)
	WDT	0.77	-0.03 (-0.24-0.18)
	TSL	0.66	0.05 (-0.16-0.25)
	CPT†	0.51	0.07 (-0.14-0.27)
	HPT†	0.7	0.04 (-0.17-0.24)
	MDT	0.66	-0.05 (-0.25-0.16)
	MPT†	0.72	0.04 (-0.17-0.24)
	MPS†	0.003	-0.3 (-0.47—0.01)
	WUR†	0.68	0.04 (-0.17-0.25)
	VDT	0.02	0.24 (0.04-0.43)
	PPT†	0.15	0.15 (-0.06-0.34)
	ULNTROM†	0.67	0.00 (-0.25-0.17)
	PPT Carpal tunnel†	0.25	-0.07 (-0.32-0.09)
	PPT Cubital tunnel†	0.6	-0.01 (-0.26-0.16)
	Serum†: IFNγ	0.44	0.08 (-13-0.29)
	IL-10	0.4	-0.1 (-0.31-0.13)
	IL-6	0.19	0.14 (-0.07-0.34)
	IL-8	0.06	0.2 (-0.02-0.39)
	TNFα	0.5	0.07 (-0.14-0.28)
	Pain score	0.55	-0.06 (-0.22-0.18)
	NDI	0.19	0.13 (-0.07-0.33)
T2 BP Signal ratio HC (pooled max)	Age	0.38	0.11 (-0.24-0.43)
	Height	0.59	0.09 (-0.26-0.42)
	Weight	0.1	0.28 (-0.07-0.57)

Correlations between T2 Brachial plexus signal ratio (maximum value from C5-C8) and clinical data. CDT, cold detection threshold; WDT, warm detection threshold, TSL, thermal sensory limen; CPT, cold pain threshold; HPT, heat pain threshold; MDT, mechanical detection threshold; MPT, mechanical pain threshold; MPS, mechanical pain sensitivity; WUR, wind up ratio; VDT, vibration detection threshold; PPT, pressure pain threshold; ULNTROM, upper limb neurodynamic test 1 (median nerve bias) range of motion; PPT, pressure pain threshold; IFNγ, Interferon gamma; IL, interleukin; TNFα, tumour necrosis factor alpha; HC, healthy control, † surrogate measures of neuroinflammation.

Supplementary table 3

Variable 1	Variable 2	p value	r value (Spearman ranks) (95% CI)
T2 DRG Signal ratio whiplash (max) †	Age	P<0.0001	0.43 (0.24-0.58)
	Height	0.16	-0.14 (0.34-0.06)
	Weight	0.25	0.12 (-0.09-0.31)
	QST z scores: CDT	0.58	0.06 (-0.15-0.26)
	WDT	0.81	0.03 (-0.18-0.23)
	TSL	0.05	0.2 (-0.00-0.39)
	CPT†	0.26	0.12 (-0.09-0.32)
	HPT†	0.23	0.12 (-0.08-0.32)
	MDT	0.53	-0.07 (-0.14-0.27)
	MPT†	0.6	0.06 (-0.15-0.26)
	MPS†	0.1	-0.06 (-0.36-0.04)
	WUR†	0.2	0.14 (-0.08-0.34)
	VDT	0.01	0.27 (0.06-0.44)
	PPT†	0.02	0.25 (-0.04-0.43)
	ULNTROM†	0.93	0.01 (-0.2-0.22)
	PPT Carpal tunnel†	0.14	-0.15 (-0.35-0.06)
	PPT Cubital tunnel†	0.21	-0.13 (-0.33-0.08)
	Serum†: IFNγ	0.29	0.11 (-1-0.32)
	IL-10	0.13	-0.17 (-0.38-0.06)
	IL-6	0.65	0.05 (-0.17-0.26)
	IL-8	0.29	0.11 (-0.1-0.32)
	TNFα	0.67	0.05 (-0.17-0.25)
	Pain score	0.98	-0.003 (-0.21-0.2)
	NDI	0.35	0.1 (-0.11-0.29)
T2 DRG Signal ratio HC (pooled max)	Age	0.0013	-0.4 (-0.65—0.07)
	Height	0.69	-0.07 (-0.4-0.28)
	Weight	0.65	0.08 (-0.27-0.4)

Correlations between T2 dorsal root ganglion signal ratio (maximum value from C5-C8) and clinical data. CDT, cold detection threshold; WDT, warm detection threshold, TSL, thermal sensory limen; CPT, cold pain threshold; HPT, heat pain threshold; MDT, mechanical detection threshold; MPT, mechanical pain threshold; MPS, mechanical pain sensitivity; WUR, wind up ratio; VDT, vibration detection threshold; PPT, pressure pain threshold; ULNTROM, upper limb neurodynamic test 1 (median nerve bias) range of motion; PPT, pressure pain threshold; IFNγ, Interferon gamma; IL, interleukin; TNFα, tumour necrosis factor alpha; HC, healthy control, † surrogate measures of neuroinflammation.
